# Characteristics, Risk Stratification, and Outcomes of Upper Gastrointestinal Bleeding in Patients Receiving Antithrombotic Therapy

**DOI:** 10.3390/biomedicines14040935

**Published:** 2026-04-20

**Authors:** Ragaey Ahmad Eid, Michael Nady Naguib, Amr Ahmed Abd El Bary, Mohamed Medhat Mohamed Zaki, Marwa O. Elgendy, Anwar M. Alnakhli, Mohammed Gamal, Mohamed Mohamed Tawfik

**Affiliations:** 1 Department of Gastroenterology, Hepatology, and Infectious Diseases (Tropical Medicine Department), Faculty of Medicine, Beni-Suef University, Beni-Suef 62511, Egypt; nadymichael64@gmail.com (M.N.N.); amrabdelbary24@gmail.com (A.A.A.E.B.); mmtawfik53@gmail.com (M.M.T.); 2 Department of Cardiology, Faculty of Medicine, Beni-Suef University, Beni-Suef 62511, Egypt; mohamed.ismail1@med.bsu.edu.eg; 3 Department of Clinical Pharmacy, Beni-Suef University Hospitals, Faculty of Medicine, Beni-Suef University, Beni-Suef 62511, Egypt; 4 Department of Clinical Pharmacy, Faculty of Pharmacy, Nahda University, Beni-Suef 62511, Egypt; 5 Department of Pharmaceutical Sciences, College of Pharmacy, Princess Nourah bint Abdulrahman University, P.O. Box 84428, Riyadh 11671, Saudi Arabia; amalnklee@pnu.edu.sa; 6 Department of Pharmaceutical Analytical Chemistry, Faculty of Pharmacy, Beni-Suef University, Alshaheed Shehata Ahmed Hegazy St., Beni-Suef 62574, Egypt; mgamalm3000@yahoo.com

**Keywords:** antithrombotic therapy, non-variceal GIT bleeding, risk stratification, rebleeding, mortality, NSAIDs

## Abstract

**Background**/**Objectives**: Non-variceal upper gastrointestinal bleeding (NVUGIB) remains a major clinical emergency, particularly among patients receiving antiplatelet or anticoagulant therapy, whose use has increased substantially in recent years. This study aimed to evaluate the clinical characteristics, endoscopic findings, risk stratification, and outcomes of NVUGIB in patients receiving antithrombotic therapy, and to compare the predictive performance of commonly used prognostic scores. **Methods**: This prospective cohort study included 89 patients receiving antithrombotic therapy who presented with NVUGIB at Beni-Suef University Hospitals between March 2023 and March 2025. Clinical presentation, laboratory findings, and endoscopic characteristics were recorded. Risk stratification was assessed using Glasgow–Blatchford (GBS), Rockall, Baylor, AIMS65, ABC, and PNED scores. The optimal cut-off values for prediction of rebleeding and mortality were determined using receiver operating characteristic (ROC) analysis and the Youden index. Area under the curve (AUC) values were reported with 95% confidence intervals. **Results**: Endoscopy revealed that peptic ulcers were the most common lesion (41/89, 46%), followed by erosive disease (27/89, 30%), with the stomach being the most frequently involved site (76.5%). Rebleeding occurred in 16 patients (18.0%), while mortality was observed in 2 patients (2.2%). The Glasgow–Blatchford score demonstrated the most consistent performance for predicting rebleeding, with an optimal cutoff value of 5.5 (derived using the Youden index), yielding 92.9% sensitivity and 78.8% specificity. For mortality prediction, AIMS65, ABC, and PNED scores showed very high AUC values, although these findings should be interpreted cautiously due to the small number of mortality events (n = 2). No statistically significant difference in rebleeding or mortality was observed between single and dual antithrombotic therapy, although patients receiving dual therapy required longer hospitalization and more transfusion units. **Conclusions**: In patients with antithrombotic-related GI bleeding, ulcers and erosions predominate, with minimal differences between single and dual therapy outcomes. Concomitant NSAID use trends toward higher mortality. Glasgow–Blatchford score offers optimal performance for both rebleeding and mortality prediction, with a cutoff of 5.5 providing excellent sensitivity (92.9%) and specificity (78.8%) for rebleeding risk assessment.

## 1. Introduction

The use of antithrombotic therapy (including antiplatelet agents and anticoagulants) has increased substantially over the past decades, particularly among elderly patients with cardiovascular diseases such as atrial fibrillation, ischemic heart disease, and thromboembolic disorders [1,2]. Although these medications play an essential role in preventing thrombotic events, their widespread use has been accompanied by an escalating incidence of gastrointestinal bleeding [3], which represents one of the most common and clinically significant adverse events associated with antithrombotic therapy [4].

Non-variceal upper gastrointestinal bleeding (NVUGIB) remains a frequent cause of hospital admission and is associated with significant morbidity, mortality, and healthcare costs [5]. Patients receiving antithrombotic therapy represent a particularly vulnerable population, as bleeding episodes may be more severe and management often requires careful balancing between hemorrhagic and thrombotic risks, including timely therapy interruption/resumption and proton pump inhibitor (PPI) prophylaxis [6].

Several risk stratification tools have been developed to predict clinical outcomes in patients with upper gastrointestinal bleeding [7,8]. Commonly used scoring systems include the Glasgow–Blatchford score (GBS), Rockall score, Baylor score, AIMS65 score, ABC score, and PNED score, which estimate the risk of adverse outcomes such as rebleeding, need for intervention, or mortality [9,10]. These tools assist clinicians in identifying high-risk patients and guiding clinical management decisions such as hospitalization, endoscopic intervention, and level of monitoring. However, most validation studies for these scores have been conducted in heterogeneous populations with upper gastrointestinal bleeding rather than specifically in patients receiving antithrombotic therapy [11].

Furthermore, many previous investigations have relied primarily on studies evaluating the comparative performance of multiple prognostic scores in patients receiving antithrombotic therapy remain limited. In addition, the influence of concomitant medications such as non-steroidal anti-inflammatory drugs (NSAIDs), as well as the anatomical distribution and type of bleeding lesions in this population, has not been sufficiently explored [12].

Therefore, this study was conducted to address these knowledge gaps.

The primary objective of this study was to evaluate the clinical characteristics, endoscopic findings, and outcomes of NVUGIB in patients receiving antithrombotic therapy.

The secondary objectives were to assess the predictive performance of commonly used prognostic scores (Glasgow–Blatchford, Rockall, Baylor, AIMS65, ABC, and PNED) for rebleeding and mortality, compare outcomes between patients receiving single versus dual antithrombotic therapy, and investigate the potential impact of concomitant NSAID use on bleeding outcomes.

## 2. Materials and Methods

### 2.1. Study Design

This prospective cohort study was conducted at Beni-Suef University Hospitals between March 2023 and March 2025. The study aimed to evaluate the clinical characteristics, endoscopic findings, risk stratification, and outcomes of non-variceal upper gastrointestinal bleeding (NVUGIB) in patients receiving antithrombotic therapy.

A post hoc power estimation suggested that approximately 200 patients would be required to detect a 20% difference in rebleeding rates between single and dual antithrombotic therapy groups with 80% power at a significance level of 0.05. The actual cohort included 89 patients, resulting in limited statistical power for subgroup analyses.

The study protocol was approved by the Ethical Committee of Beni-Suef University Hospitals (Approval No: FMBSUREC/12022023/ Naguib).

### 2.2. Study Population

The study included adult patients (≥18 years) admitted with upper gastrointestinal bleeding while receiving antithrombotic therapy, including antiplatelet agents or anticoagulants.

#### 2.2.1. Inclusion Criteria

Adult patients aged ≥18 years; presentation with symptoms of upper gastrointestinal bleeding (e.g., hematemesis or melena); current use of antithrombotic therapy confirmed through medical history and active prescription records and provision of written informed consent.

#### 2.2.2. Exclusion Criteria

Patients not receiving antithrombotic therapy prior to the bleeding episode; patients younger than 18 years; variceal gastrointestinal bleeding; lower gastrointestinal bleeding; obscure gastrointestinal bleeding and patients unable or unwilling to provide informed consent.

### 2.3. Patient Evaluation

To improve clarity and reproducibility, patient evaluation variables were categorized into several domains.

#### 2.3.1. Demographic Variables

Demographic information including age and sex was recorded for all patients.

#### 2.3.2. Clinical Characteristics

A detailed clinical history was obtained, including presenting symptoms (hematemesis, melena, or hematochezia), smoking status, alcohol consumption, and indication for antithrombotic therapy.

Baseline clinical assessment included vital signs, cardiovascular examination, abdominal examination, and rectal examination to evaluate the severity of bleeding and detect potential complications.

##### Management of Antithrombotic Therapy

In patients presenting with active gastrointestinal bleeding, antithrombotic therapy was temporarily withheld upon hospital admission. Decisions regarding the timing of therapy resumption were made on an individualized basis by a multidisciplinary team including gastroenterologists and cardiologists, taking into account the severity of bleeding, endoscopic findings, and the patient’s underlying thrombotic risk.

All patients received proton pump inhibitor (PPI) therapy during hospitalization according to institutional protocols for the management of upper gastrointestinal bleeding.

#### 2.3.3. Comorbidities

Information regarding comorbid conditions was collected, including diabetes mellitus, hypertension, ischemic heart disease, atrial fibrillation, chronic kidney disease, liver cirrhosis, heart failure, stroke, and malignancies.

#### 2.3.4. Concomitant Medications

Details of antithrombotic therapy were documented, including drug class, specific medication, dosage, and duration of treatment.

Concomitant medications such as non-steroidal anti-inflammatory drugs (NSAIDs) and other potentially ulcerogenic drugs were also recorded.

#### 2.3.5. Clinical Outcomes

Clinical outcomes evaluated during hospitalization included:

- Rebleeding events.
- Length of hospital stay.
- Blood transfusion requirements.
- Need for therapeutic endoscopic intervention.
- In-hospital mortality.

### 2.4. Laboratory and Radiological Investigations

Laboratory investigations were performed according to the institutional protocol for patients presenting with gastrointestinal bleeding [13]. Baseline investigations included:

- Complete blood count.
- Renal function tests (serum creatinine, urea, blood urea nitrogen).
- Liver function tests.
- Electrolytes.
- Coagulation profile.

Repeat laboratory testing was performed when clinically indicated during hospitalization.

Radiological investigations were performed when clinically indicated and included:

- Abdominopelvic ultrasound to evaluate liver cirrhosis or portal hypertension.
- Echocardiography in patients with suspected or known atrial fibrillation.
- Venous or arterial duplex imaging when vascular disease was suspected.

### 2.5. Endoscopic Evaluation

All patients underwent upper gastrointestinal endoscopy to identify the source of bleeding [14]. Endoscopic findings were documented including:

- Type of lesion (ulcer, erosion, malignancy, or other lesions).
- Anatomical location of bleeding (esophagus, stomach, or duodenum).
- Forrest classification for bleeding peptic ulcers.

Therapeutic endoscopic interventions were performed when indicated.

### 2.6. Risk Stratification

Risk stratification was performed using several established scoring systems for upper gastrointestinal bleeding [15]:

- Glasgow–Blatchford score (GBS).
- Rockall score.
- Baylor score.
- AIMS65 score.
- ABC score.
- PNED score.

These scoring systems incorporate clinical, laboratory, and endoscopic variables to estimate the risk of adverse outcomes such as rebleeding [16], need for intervention, or mortality [17].

### 2.7. Definition of Rebleeding

Rebleeding was defined as recurrent upper gastrointestinal bleeding occurring after initial successful hemostasis within 7 days of the index bleeding episode. Rebleeding was diagnosed when one or more of the following criteria were present [18]:

- Recurrence of hematemesis or melena.
- A decrease in hemoglobin ≥2 g/dL after initial stabilization.
- Development of hemodynamic instability requiring clinical intervention.
- Requirement for repeat endoscopic therapy or additional blood transfusion.

### 2.8. Statistical Analysis

Data were analyzed using Statistical Package for the Social Sciences (SPSS) software (Version 29.0, IBM Corp., Armonk, NY, USA).

Continuous variables were expressed as mean ± standard deviation (SD), while categorical variables were presented as frequencies and percentages.

Comparisons between groups were performed using:

- Student’s *t*-test or Mann–Whitney U test for continuous variables.
- Chi-square test or Fisher’s exact test for categorical variables.

Receiver operating characteristic (ROC) curve analysis was used to evaluate the predictive performance of the different scoring systems for rebleeding and mortality. The area under the curve (AUC) was calculated with corresponding 95% confidence intervals, and optimal cut-off values were determined using the Youden index.

Multivariable regression analysis was considered; however, the relatively small number of outcome events (particularly mortality (n = 2)) limited the number of variables that could be reliably included in a multivariable model without violating recommended statistical assumptions. Therefore, analyses were restricted to univariate comparisons.

Because the number of mortality events was extremely limited, ROC analyses for mortality prediction were considered exploratory and should be interpreted with caution due to potential statistical instability and risk of overfitting.

A *p*-value < 0.05 was considered statistically significant.

## 3. Results

### 3.1. Patient Enrollment

A total of 2000 patients presenting with gastrointestinal bleeding were initially assessed in the endoscopy unit during the study period. Among them, 1900 patients were excluded because they were not receiving antithrombotic therapy.

Of the remaining 100 patients receiving antithrombotic therapy, 5 patients were diagnosed with obscure gastrointestinal bleeding and 6 with lower gastrointestinal bleeding and were therefore excluded from further analysis.

Consequently, 89 patients with upper gastrointestinal bleeding were included in the final study cohort.

Endoscopic findings revealed that ulcers were the most common cause of bleeding (n = 41), followed by erosions (n = 27). Malignancy-related bleeding was observed in three cases, including one case of duodenal adenocarcinoma and two cases of gastric carcinoma.

**Figure 1:**
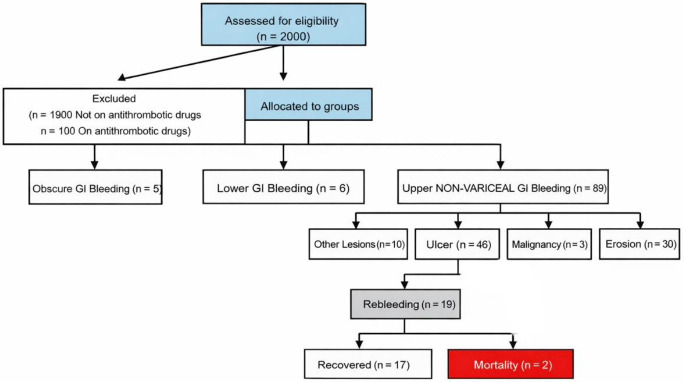
Consort flow diagram of the eligible cases enrolment in the endoscopy unit.

Among patients with ulcer-related bleeding, rebleeding occurred in 16 patients (18.0%), of whom 14 recovered after appropriate management, while two patients with Forrest class IA and IB ulcers died due to severe uncontrolled bleeding despite intensive resuscitative measures (Figure 1).

### 3.2. Demographic Characteristics and Comorbidities

The mean age of the study population was 60.99 ± 15.51 years (range: 17–90 years). Males represented the majority of the cohort (58/89, 65.2%), while females accounted for 31 patients (34.8%).

The cohort exhibited a high prevalence of cardiovascular and metabolic comorbidities. The most frequent comorbid conditions included hypertension (30%), ischemic heart disease (33%), diabetes mellitus (20%), atrial fibrillation (14%), and stroke (16%).

Regarding indications for antithrombotic therapy, ischemic heart disease was the most common indication (n = 33, 37.1%), followed by venous thromboembolism prophylaxis (n = 25, 28.1%).

Detailed demographic and clinical characteristics are presented in Table 1.

### 3.3. Distribution of Antithrombotic Therapy

Antithrombotic therapy was categorized into single antiplatelet therapy, anticoagulant therapy, and dual antithrombotic therapy.

The majority of patients received single antiplatelet therapy (n = 55, 61.8%), most commonly aspirin (n = 50, 56.2%).

Anticoagulant therapy was used in 21 patients (23.6%), including warfarin (n = 9), direct oral anticoagulants (n = 10), and low-molecular-weight heparin (n = 2).

Dual antithrombotic therapy was observed in 13 patients (14.6%), consisting of dual antiplatelet therapy (n = 5) or combined antiplatelet and anticoagulant therapy (n = 8).

The distribution of antithrombotic therapy is summarized in Table 2.

### 3.4. Endoscopic Findings

#### 3.4.1. Lesion Type

Endoscopic examination revealed that peptic ulcers were the most common lesion type (41/89, 46%), followed by erosive disease (27/89, 30%).

Malignancy-related bleeding accounted for three cases, while other lesions included gastritis, gastric antral vascular ectasia (GAVE), and reflux esophagitis.

#### 3.4.2. Anatomical Location of Bleeding

Regarding the anatomical distribution of lesions, the stomach was the most frequently affected site (76.5%), followed by the duodenum (13.8%) and the esophagus (9.7%).

#### 3.4.3. Forrest Classification

Among patients with peptic ulcer bleeding, Forrest class III ulcers were the most frequent (48%), followed by Forrest class IIB (26%), Forrest IIC (14%), and Forrest IIA (8%). Active bleeding ulcers (Forrest IA and IB) accounted for a small proportion of cases (2% each).

Detailed lesion characteristics and anatomical distribution are presented in Table 3.

### 3.5. Comparison Between Monotherapy and Dual Antithrombotic Therapy

Patients were categorized into monotherapy (single antithrombotic agent) and dual antithrombotic therapy groups.

No statistically significant differences were observed between the two groups in terms of rebleeding or mortality.

Rebleeding occurred in 12 patients receiving monotherapy and 4 patients receiving dual therapy (*p* = 0.919). Mortality was observed in two patients in the monotherapy group, while no deaths occurred in the dual therapy group (*p* = 0.421).

However, patients receiving dual antithrombotic therapy experienced significantly longer hospital stays compared with those receiving monotherapy (7.79 ± 5.53 vs. 5.89 ± 2.95 days, *p* = 0.032) (Table 4 and Table 5).

Similarly, the number of packed red blood cell units transfused was significantly higher in the dual therapy group (2.04 ± 1.43 vs. 1.17 ± 1.69 units, *p* = 0.025).

These findings suggest that although dual therapy was not associated with increased rebleeding or mortality, it was associated with greater healthcare resource utilization.

### 3.6. Impact of Concomitant NSAID Use

Patients who reported concomitant NSAID use showed higher rebleeding rates compared with those who did not use NSAIDs (18.8% vs. 8.3%), although this difference did not reach statistical significance (*p* = 0.203).

Similarly, mortality appeared higher among patients using NSAIDs (50.0% vs. 1.1%), but this finding did not reach statistical significance (*p* = 0.057) and should therefore be interpreted cautiously due to the small number of mortality events (Table 5).

### 3.7. Management of Rebleeding

The majority of patients (81%) experienced no further bleeding after initial management and required no additional interventions.

Among patients who experienced recurrent bleeding, management strategies included medical therapy, repeat endoscopic hemostasis using hemoclips or hemostatic spray, argon plasma coagulation, and surgical intervention when necessary.

The management approaches for rebleeding are summarized in Table 6.

### 3.8. Performance of Risk Stratification Scores for Predicting Rebleeding

Receiver operating characteristic (ROC) curve analysis was performed to evaluate the predictive performance of the Glasgow–Blatchford score (GBS), Rockall score, and Baylor score for predicting rebleeding.

Both the GBS (AUC = 0.764) and Rockall score (AUC = 0.761) demonstrated comparable predictive accuracy, while the Baylor score showed lower predictive ability (AUC = 0.660).

The optimal cutoff value for GBS was 5.5, which provided 92.9% sensitivity and 78.8% specificity for predicting rebleeding.

**Figure 2:**
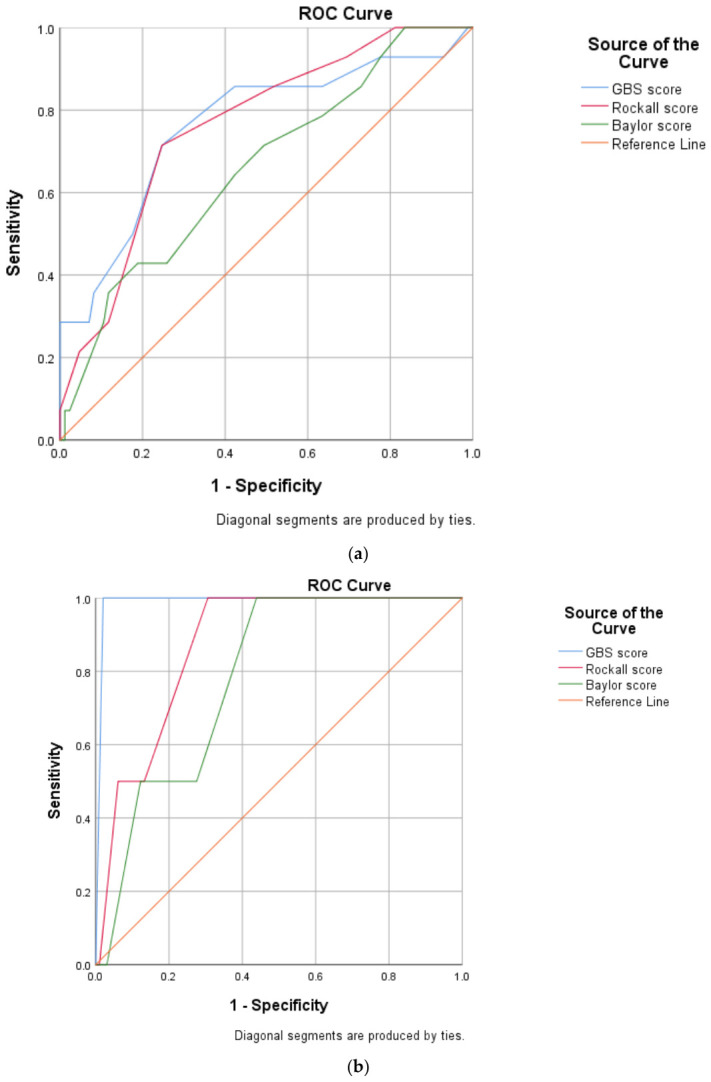

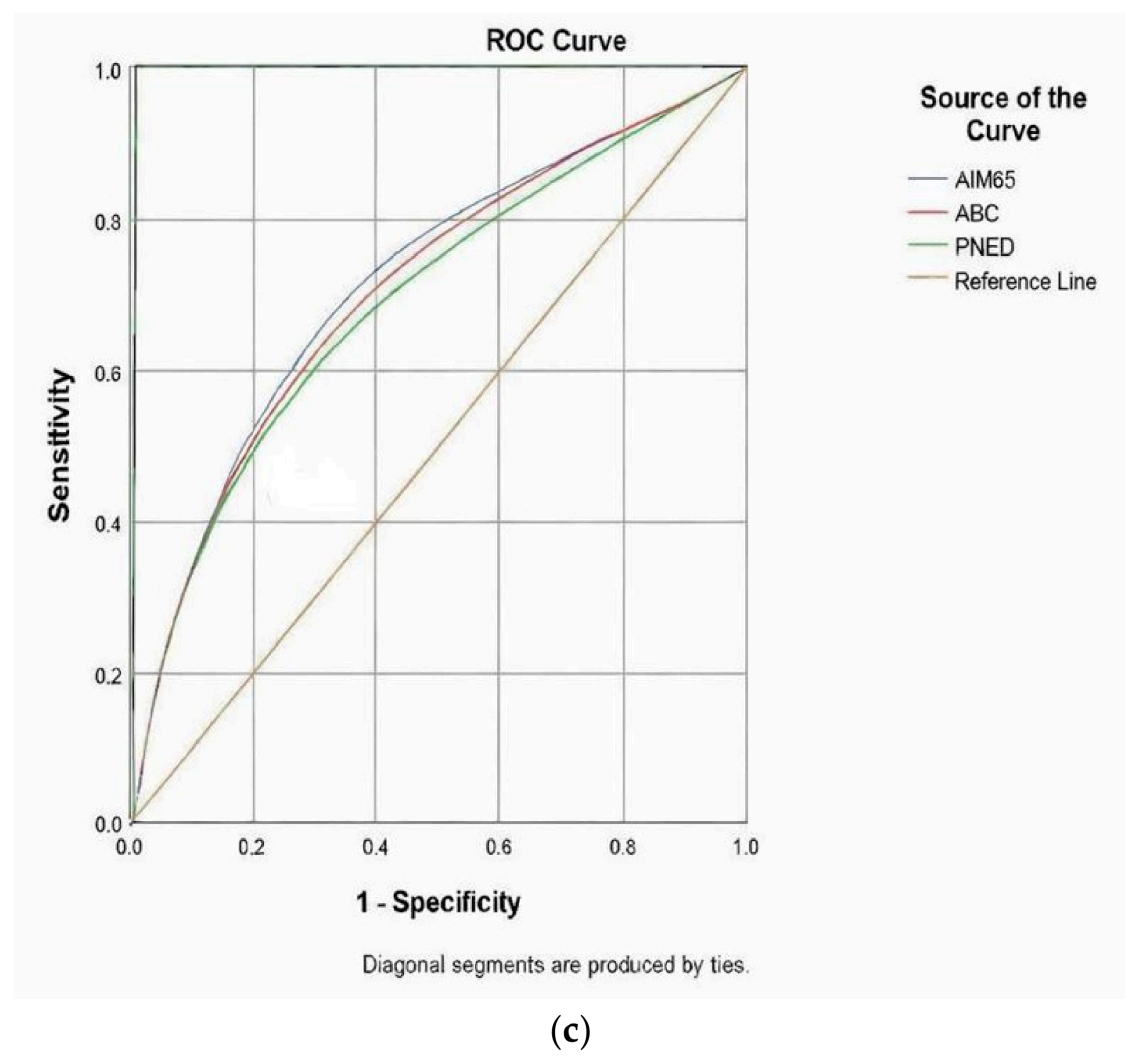
(**a**) ROC curves for rebleeding prediction using GBS (AUC 0.764), Rockall (0.761), and Baylor (0.660). Optimal GBS cutoff 5.5 (sens 92.9%, spec 78.8%). Mortality ROCs omitted (n = 2 events preclude stability). (**b**) Roc Curve for predicting mortality based on Glasgow–Blatchford Score (GBS), Rockall Score & Baylor Score. (**c**) Roc Curve for predicting mortality based on AIM65, ABC, and PRED scores.

Baylor Score shows lower predictive capacity at comparable cutoff levels, as shown in Figure 2a.

### 3.9. Exploratory ROC Analysis for Mortality Prediction

Because only two mortality events occurred in the study cohort, ROC curve analyses for mortality prediction must be interpreted with extreme caution.

In this exploratory analysis, the Glasgow–Blatchford score showed the highest apparent discrimination (AUC = 0.990), followed by the Rockall score (AUC = 0.872) and the Baylor score (AUC = 0.783).

However, these estimates are likely influenced by statistical instability related to the extremely small number of outcome events and therefore should be considered exploratory rather than definitive measures of predictive performance.

The Baylor Score at 8.5 maintains high sensitivity (100%) but has lower specificity (49.0%), as shown in Figure 2b.

### 3.10. Exploratory ROC Analysis Using AIMS65, ABC, and PNED Scores

ROC analyses were also performed to evaluate the ability of AIMS65, ABC, and PNED scores to predict mortality.

All three scoring systems demonstrated near-perfect AUC values (0.995).

Given the presence of only two mortality events in the entire cohort, these findings likely reflect statistical overfitting rather than true predictive accuracy and should therefore be interpreted cautiously as exploratory observations (Figure 2c).

## 4. Discussion

The present study evaluated the clinical characteristics, endoscopic findings, and prognostic performance of several risk stratification scores in patients presenting with non-variceal upper gastrointestinal bleeding (NVUGIB) while receiving antithrombotic therapy. The main findings of the present study were that peptic ulcer disease represented the most frequent cause of bleeding, the Glasgow–Blatchford score demonstrated the most consistent performance for predicting rebleeding, and no statistically significant difference in major outcomes was observed between single and dual antithrombotic therapy, although dual therapy was associated with longer hospitalization and greater transfusion requirements.

Antithrombotic medications are among the most commonly prescribed drugs worldwide [19,20,21,22]. Their increasing use in aging populations with cardiovascular disease has contributed to a rising incidence of gastrointestinal bleeding associated with these therapies [23].

In our cohort, the mean age of patients was 60.99 years, reflecting the fact that antithrombotic therapy is most commonly prescribed in older populations. Older age is a well-recognized risk factor for gastrointestinal bleeding, largely due to age-related mucosal vulnerability, polypharmacy, and the high prevalence of comorbidities [24]. Additionally, elderly patients frequently receive multiple medications such as antiplatelets [25], anticoagulants, and NSAIDs, which may further increase bleeding risk [26]. Age-related pharmacokinetic and pharmacodynamic changes may also contribute to unstable anticoagulation control or altered drug metabolism, thereby increasing the risk of bleeding complications [27].

In the present study, peptic ulcers were identified as the most common bleeding source (46%), followed by erosive lesions (30%), with the stomach representing the most frequently affected anatomical site (76.5%). These findings are consistent with previous reports demonstrating that ulcer disease remains the predominant cause of NVUGIB in patients receiving antithrombotic therapy.

Our observation that ulcers were particularly common among patients receiving aspirin and direct oral anticoagulants aligns with the findings of Choe et al., who reported differences in bleeding patterns between vitamin K antagonists and newer oral anticoagulants [28]. Similarly, Uotani et al. demonstrated that both clopidogrel and low-dose aspirin may induce significant gastric mucosal injury [29].

The mechanisms underlying antithrombotic-associated gastrointestinal injury vary between drug classes. Aspirin may cause direct mucosal injury through disruption of the gastric mucus barrier while also inhibiting cyclooxygenase-1 (COX-1), leading to reduced prostaglandin synthesis and impaired mucosal protection [30]. Clopidogrel may impair ulcer healing by inhibiting platelet-derived growth factors involved in mucosal repair [31]. Direct oral anticoagulants may increase bleeding risk through inhibition of thrombin or factor Xa while potentially causing local mucosal irritation [32].

Regarding antithrombotic therapy regimens, our analysis did not demonstrate significant differences in rebleeding or mortality between patients receiving single versus dual antithrombotic therapy. However, patients receiving dual therapy required significantly longer hospital stays and higher numbers of transfused packed red blood cell units. These findings suggest that although dual antithrombotic therapy may not necessarily increase the risk of catastrophic outcomes such as mortality in this cohort, it may be associated with greater clinical complexity and increased healthcare resource utilization.

These findings are partly consistent with the results reported by Dakroub et al., who observed comparable bleeding outcomes between anticoagulant monotherapy and combination therapy in patients with atrial fibrillation presenting with gastrointestinal bleeding [33]. Similarly, van Diepen et al. reported comparable bleeding outcomes between dual antiplatelet therapy and aspirin monotherapy in patients undergoing coronary artery bypass grafting [34].

One possible explanation for the increased resource utilization observed in the dual therapy group is that combined antithrombotic regimens may result in more complex bleeding episodes requiring prolonged monitoring and supportive care, even when the risk of catastrophic outcomes such as mortality is not significantly increased.

In the present study, patients using concomitant NSAIDs demonstrated higher rebleeding rates compared with those who did not use NSAIDs, although this difference did not reach statistical significance. A higher proportion of mortality events was also observed among NSAID users; however, this observation must be interpreted with caution due to the extremely small number of mortality events recorded in the cohort.

Previous studies have consistently demonstrated that NSAIDs significantly increase the risk of gastrointestinal bleeding through both systemic and topical mechanisms. NSAIDs inhibit COX-1–mediated prostaglandin synthesis, reducing mucosal protection and impairing platelet aggregation, while also exerting direct topical effects on the gastric mucosa [35].

Our findings are broadly consistent with previous studies reporting increased bleeding risk among NSAID users. For example, Aoki et al. identified NSAID use as an independent risk factor for rebleeding [36], while Lanas et al. reported that NSAIDs substantially increase the risk of both upper and lower gastrointestinal bleeding [5].

Our two mortalities had occurred in patients with Forrest class 1A/1B ulcers, confirming lesion severity as the key determinant of mortality in antithrombotic-related bleeding [37], where endoscopist expertise significantly impacts outcomes, with specialized centres achieving better hemostasis for high-risk lesions [38]. Our resources, which align with guidelines emphasizing facility capabilities in managing severe UGIB [39], support protocolized pathways that prioritize urgent endoscopy, dual therapy, and early ICU admission, which may be more impactful than medication adjustments alone [40].

Analysis of our data demonstrated that the Glasgow Blatchford score and Rockall score showed similar predictive accuracy for rebleeding, whereas the Baylor score showed lower predictive performance. The optimal GBS cutoff value for predicting rebleeding in our cohort was 5.5, which provided high sensitivity and acceptable specificity.

ROC analyses were also performed to explore the ability of several scoring systems to predict mortality. However, only two mortality events occurred in the entire cohort [41]. This extremely limited number of outcome events substantially reduces the statistical stability of ROC analysis and increases the likelihood of artificially inflated discrimination estimates [42,43]. Consequently, the very high AUC values observed for some scoring systems should be interpreted cautiously and considered exploratory rather than definitive evidence of predictive performance [44].

However, our findings contrast with Chang et al. [45] and Nakamura et al. [46] (both found AIMS65, not GBS, predicted mortality), Yang et al. and Budimir et al. (pRS more effective than GBS for death prediction) [47,48], while Ramaekers et al. found GBS more accurate for both intervention and 30-day mortality [49], and Laursen et al. found GBS poor for mortality prediction [50].

These variations in predictive accuracy across studies likely stem from several key factors: diverse patient demographics and comorbidity profiles [51], methodological differences as rebleeding and mortality endpoints in-hospital vs. 30-day vs. 90-day) [52], variable treatment protocols [53], and institutional characteristics [54]. Most importantly, each scoring system emphasizes different clinical parameters [55]. This explains why no single scoring system demonstrates universal superiority, with their relative performance varying based on the specific population characteristics and outcome measures prioritized in each study.

The management of antithrombotic therapy in patients presenting with gastrointestinal bleeding remains clinically challenging. Temporary interruption of antithrombotic therapy is often necessary to achieve hemostasis; however, prolonged discontinuation may increase the risk of thromboembolic complications. Current guidelines, therefore, recommend individualized decisions regarding the interruption and resumption of antithrombotic therapy based on the severity of bleeding and the patient’s underlying thrombotic risk.

In addition, proton pump inhibitor (PPI) therapy plays a critical role in reducing the risk of recurrent bleeding in patients receiving antiplatelet or anticoagulant therapy, particularly among individuals with additional risk factors such as prior peptic ulcer disease or concomitant NSAID use. Optimization of antithrombotic strategies and avoidance of unnecessary combination therapy may further reduce bleeding risk while maintaining adequate thrombotic protection. Recent literature has emphasized the importance of individualized antithrombotic regimens aimed at minimizing bleeding complications without compromising cardiovascular outcomes [56].

However, interpretation of mortality prediction analyses must be approached with caution, as only two mortality events occurred in the study cohort. Although very high AUC values were observed for several scores, including AIMS65, ABC, and PNED, these findings are likely influenced by statistical instability and potential overfitting due to the extremely low number of outcome events.

Study Limitations.

This study has several limitations that should be acknowledged. First, the sample size was relatively small (n = 89), which limits the statistical power for subgroup analyses. Second, the number of mortality events was extremely low (n = 2), which significantly limits the reliability of mortality prediction analyses and increases the risk of statistical instability in ROC curve estimates. Third, the study was conducted at a single tertiary care center, which may limit the generalizability of the findings.

In addition, multivariable regression analysis was not performed because the limited number of outcome events would not support reliable multivariable modeling according to recommended statistical standards. Larger multicenter studies are therefore needed to validate these findings and to better evaluate independent predictors of adverse outcomes.

Study Strengths.

Despite these limitations, the study has several strengths. The prospective study design allowed systematic data collection and reduced recall bias. Furthermore, the simultaneous evaluation of multiple risk stratification scores provides useful comparative insights into their performance in patients receiving antithrombotic therapy. The study also reflects real-world clinical practice, where patients frequently present with complex combinations of comorbidities and medications.

## 5. Conclusions

In patients presenting with non-variceal upper gastrointestinal bleeding while receiving antithrombotic therapy, peptic ulcer disease remains the most common underlying cause of bleeding, with the stomach representing the most frequently affected anatomical site.

Among the evaluated prognostic tools, the Glasgow–Blatchford score demonstrated the most consistent performance for predicting rebleeding, supporting its use in early risk stratification of patients presenting with antithrombotic-associated gastrointestinal bleeding.

No significant differences in rebleeding or mortality were observed between single and dual antithrombotic therapy in this cohort, although patients receiving dual therapy required longer hospitalization and greater transfusion support, suggesting increased healthcare resource utilization.

Because of the extremely small number of mortality events observed in this study, conclusions regarding mortality prediction using existing scoring systems remain limited. Larger prospective multicenter studies are required to validate these findings and to further evaluate optimal strategies for risk stratification and management of gastrointestinal bleeding in patients receiving antithrombotic therapy.

## Figures and Tables

**Table 1:** Demographic characteristics, comorbidities of the studied patients.

Characteristic	Mean ± (Std. Dev.)
Age (years)	60.99 ± 15.51
Gender N (%)	Male: 58 (65.2%)
	Female: 31 (34.8%)
Comorbidities	n (%)
Diabetes mellitus	20 (22.5%)
Hypertension	30 (33.7%)
Ischemic heart disease	33 (37.1%)
Atrial fibrillation	14 (15.7%)
Liver Cirrhosis	7 (7.9%)
CKD or ESRD	9 (10.1%)
Stroke	16 (18.0%)

**Table 2:** Distribution of Antithrombotic Therapy by Category and Specific Medication.

Antithrombotic Category	Number of Patients	Percentage (%)
Single Antiplatelet therapy (total)	55	61.8
- Aspirin	50	56.2
- Clopidogrel	4	4.5
- Other antiplatelet agents	1	1.1
Anticoagulant therapy (total)	21	23.6
- Vitamin K antagonists (Warfarin)	9	10.1
- Direct Oral Anticoagulants (total)	10	11.2
- Low Molecular Weight Heparin	2	2.2
Dual therapy (total)	13	14.6
- Dual antiplatelet therapy	5	5.6
- Antiplatelet + anticoagulant	8	9.0
Total	89	100.0

**Table 3:** Distribution of Lesion Types Among Patients on Antithrombotic Medications & Distribution of Lesion Sites across Upper GI Bleeding Cases.

	Medication				
	Aspirin	Clopidogrel	Warfarin	New Oral anticoagulant	LMWH
Lesion type	N (%)	N (%)	N (%)	N (%)	N (%)
Erosion	20 (28.6)	0 (0.0)	5 (45.5)	3 (30.0)	2 (100.0)
Ulcer	39 (55.7)	1 (100.0)	4 (36.4)	6 (60.0)	0 (0.0)
Malignancy	1 (1.4)	0 (0.0)	1 (9.1)	1 (10.0)	0 (0.0)
Other	10 (14.3)	0 (0.0)	1 (9.1)	0 (0.0)	0 (0.0)
	Site of lesion (n = 89)				
	Esophagus N (%)	Stomach N (%)	Duodenum N (%)		
Erosion	2 (7.0)	25 (83.0)	3 (10.0)		
Ulcer	6 (12.0)	35 (70.0)	9 (18.0)		
Malignancy	1 (33.3)	1 (33.3)	1 (33.3)		
Gastritis	0 (0.0)	7 (100.0)	0 (0.0)		
GAVE	-	4 (100.0)	-		

**Table 4:** Impact of Monotherapy vs. Dual Therapy on Hospital Stay and Blood Transfusion Requirements.

	Number of Medications	N	Mean	Std. Deviation	*p*-Value
Length of hospital stay	Monotherapy	66	5.89	2.951	0.032 *
	Double therapy	23	7.79	5.532	
Units of Packed RBCs transfused	Monotherapy	66	1.17	1.692	0.025 *
	Double therapy	23	2.04	1.429	

* Refers to significance.

**Table 5:** Rebleeding/Mortality by Antithrombotic Regimen and NSAID Use.

		Monotherapy		Double Therapy		*p*-Value
		Count (66)	Row N %	Count (23)	Row N %	
Rebleeding	No	54	81.8%	19	82.6%	0.919
	Yes	12	18.2%	4	17.4%	
Mortality	No	64	97%	23	100%	0.421
	Yes	2	3%	0	0.0%	
		NSAIDS intake				*p*-value
		No intake		Yes		
		Count (79)	Row N %	Count (10)	Row N %	
Rebleeding	No	72	91.1%	7	70%	0.203
	Yes	7	8.9%	3	30%	
Mortality	No	78	98.7%	9	90%	0.057
	Yes	1	1.3%	1	10%	

**Table 6:** Management of Gastrointestinal Rebleeding.

		Frequency	Percent
Valid	No Rebleeding (No intervention)	81	81.0
	2nd section of argon plasma coagulation	1	1.0
	Hemocliping	1	1.0
	hemostatic spray	1	1.0
	Management of rebleeding	1	1.0
	stopped on medical therapy	11	11.0
	surgical debulking	1	1.0
	surgical hemorrhoidectomy	1	1.0

## Data Availability

Raw data supporting this study’s findings are available from the corresponding authors upon reasonable request.

## References

[1] Gralnek I.M., Stanley A.J., Morris A.J., Camus M., Lau J., Lanas A., Laursen S.B., Radaelli F., Papanikolaou I.S., Gonçalves T.C. (2021). Endoscopic diagnosis and management of nonvariceal upper gastrointestinal hemorrhage (NVUGIH): European Society of Gastrointestinal Endoscopy (ESGE) Guideline—Update 2021. Endoscopy.

[2] Laine L., Barkun A.N., Saltzman J.R., Martel M., Leontiadis G.I. (2021). ACG clinical guideline: Upper gastrointestinal and ulcer bleeding. Off. J. Am. Coll. Gastroenterol. ACG.

[3] Shaban M., Elgendy M.O., Fahmy A.M., Khalil D.M., El-Gendy A.O., Mahmoud T.M., Abdelrahim M.E. (2022). The Outcomes of COVID-19 Patients with Spontaneous Intracerebral Hemorrhage Comorbidity and the Efficacy of Enoxaparin in Decreasing the Mortality Rate in Them: Single Egyptian Center Report. J. Pers. Med..

[4] Di Minno A., Spadarella G., Prisco D., Scalera A., Ricciardi E., Di Minno G. (2015). Antithrombotic drugs, patient characteristics, and gastrointestinal bleeding: Clinical translation and areas of research. Blood Rev..

[5] Lanas Á., Carrera-Lasfuentes P., Arguedas Y., García S., Bujanda L., Calvet X., Ponce J., Perez-Aísa Á., Castro M., Muñoz M. (2015). Risk of upper and lower gastrointestinal bleeding in patients taking nonsteroidal anti-inflammatory drugs, antiplatelet agents, or anticoagulants. Clin. Gastroenterol. Hepatol..

[6] Di Minno M.N.D., Ambrosino P., Di Minno A., Tremoli E., Di Minno G. (2017). The risk of gastrointestinal bleeding in patients receiving dabigatran etexilate: A systematic review and meta-analysis of the literature. Ann. Med..

[7] Battistoni I., Francioni M., Morici N., Rubboli A., Podda G.M., Pappalardo A., Abdelrahim M.E., Elgendy M.O., Elgendy S.O., Khalaf A.M. (2022). Pre-and in-hospital anticoagulation therapy in coronavirus disease 2019 patients: A propensity-matched analysis of in-hospital outcomes. J. Cardiovasc. Med..

[8] Fahmy A., Elgendy M., Khalaf A., Abdelrahman M., Abdelrahim M., El-Gendy A. (2022). COVID-19 patients with hepatic complications during the third wave of pandemic in Egypt. J. Clin. Nurs. Res..

[9] Mohamed A.A., Elkholy M.M., Mangoud O.O., Ibrahim A.R., Elgendy M.O., Abdel Fattah A.M. (2024). Psychometric Hepatic Encephalopathy Tests and Electroencephalogram Results Among Cirrhotic Patients. Medicina.

[10] Farag M.H., Shaaban M.H., Abdelkader H., Al Fatease A., Elgendy S.O., Okasha H.H. (2025). Predictors of Complications in Radiofrequency Ablation for Hepatocellular Carcinoma: A Comprehensive Analysis of 1000 Cases. Medicina.

[11] Pannach S., Goetze J., Marten S., Schreier T., Tittl L., Beyer-Westendorf J. (2017). Management and outcome of gastrointestinal bleeding in patients taking oral anticoagulants or antiplatelet drugs. J. Gastroenterol..

[12] Cangemi D.J., Krill T., Weideman R., Cipher D.J., Spechler S.J., Feagins L.A. (2017). A comparison of the rate of gastrointestinal bleeding in patients taking non-vitamin K antagonist oral anticoagulants or warfarin. Off. J. Am. Coll. Gastroenterol. ACG.

[13] Sabry R.M., Hamad O., Khalil H.E.M., Mohammed S.I., Eid R.A., Hosny H. (2025). The role of multifocal visual evoked potential in detection of minimal hepatic encephalopathy in patients with compensated liver cirrhosis. BMC Neurol..

[14] Afonso J., Saraiva M.M., Ferreira J.P.S., Cardoso H., Ribeiro T., Andrade P., Parente M., Jorge R.N., Macedo G. (2022). Automated detection of ulcers and erosions in capsule endoscopy images using a convolutional neural network. Med. Biol. Eng. Comput..

[15] Dhir V., Shah R. (2019). Scoring systems for upper gastrointestinal bleeding: Which one scores better?. Indian J. Gastroenterol..

[16] Mokhtare M., Bozorgi V., Agah S., Nikkhah M., Faghihi A., Boghratian A., Shalbaf N., Khanlari A., Seifmanesh H. (2016). Comparison of Glasgow-Blatchford score and full Rockall score systems to predict clinical outcomes in patients with upper gastrointestinal bleeding. Clin. Exp. Gastroenterol..

[17] Hyett B.H., Abougergi M.S., Charpentier J.P., Kumar N.L., Brozovic S., Claggett B.L., Travis A.C., Saltzman J.R. (2013). The AIMS65 score compared with the Glasgow-Blatchford score in predicting outcomes in upper GI bleeding. Gastrointest. Endosc..

[18] Saeed Z.A., Ramirez F.C., Hepps K.S., Cole R.A., Graham D.Y. (1995). Prospective validation of the Baylor bleeding score for predicting the likelihood of rebleeding after endoscopic hemostasis of peptic ulcers. Gastrointest. Endosc..

[19] Hamied A.M.A., Ahmed H.M., Eldahshan D.H., Morgan D.S., Meged A.M.A.A., Elgendy M.O., Imam M.S., Alotaibi T.A., Alotaibi M.M., Alotaibi M.T. (2023). Association of Bone Disorder and Gene Polymorphism of PPAR-γ Pro12 Ala in Egyptian Children with β-Thalassemia. Thalass. Rep..

[20] Abdou L.M., El-Gendy A.O., Elgendy M.O., Gad R.A., Elgendy S.O., Eid R.A., Sayed A.M., Mahmoud T.M. (2022). The Impact of Combining Cefepime or Ceftazidime with Steroidal and Anticoagulant Therapy in the Treatment of COVID-19 Patients. NeuroQuantology.

[21] Khalaf A.M., Elgendy M.O., Fahmy A.M., El Gendy S.O., El-Gendy A.O., Abdelrahman M.A., El-Bahrawy A.H., Elsisi A.M.M., Shafiq S.N. (2024). Relation between ABO and RhD and prevalence and severity of COVID-19 disease. Int. J. Clin. Med. Res..

[22] Zaki A., Elgendy M.O., Abdelrahman M.A., Ali H., Khalil E.M., Hassan M., Fahmy A.M., Gad R.A., Salem H.F. (2023). The efficacy of using different antibiotics to prevent maternal surgical site infections in COVID-19-infected cases. Eur. Chem. Bull.

[23] Troy A., Anderson T.S. (2021). National trends in use of and spending on oral anticoagulants among US Medicare beneficiaries from 2011 to 2019. JAMA Health Forum.

[24] Marinigh R., Lip G.Y., Fiotti N., Giansante C., Lane D.A. (2010). Age as a risk factor for stroke in atrial fibrillation patients: Implications for thromboprophylaxis. J. Am. Coll. Cardiol..

[25] Chi T.-Y., Zhu H.-M., Zhang M. (2018). Risk factors associated with nonsteroidal anti-inflammatory drugs (NSAIDs)-induced gastrointestinal bleeding resulting on people over 60 years old in Beijing. Medicine.

[26] Bajorek B. (2011). A review of the safety of anticoagulants in older people using the medicines management pathway: Weighing the benefits against the risks. Ther. Adv. Drug Saf..

[27] Martin A.-C., Benamouzig R., Gouin-Thibault I., Schmidt J. (2023). Management of gastrointestinal bleeding and resumption of oral anticoagulant therapy in patients with atrial fibrillation: A multidisciplinary discussion. Am. J. Cardiovasc. Drugs.

[28] Choe A.R., Moon C.M., Tae C.H., Chun J., Bang K.B., Lee Y.J., Lee H.S., Jung Y., Park S.C., Koo H.S. (2021). Characteristics, location, and clinical outcomes of gastrointestinal bleeding in patients taking new oral anticoagulants compared to vitamin K antagonists. J. Clin. Med..

[29] Uotani T., Sugimoto M., Nishino M., Kodaira C., Yamade M., Sahara S., Yamada T., Osawa S., Sugimoto K., Tanaka T. (2012). Ability of rabeprazole to prevent gastric mucosal damage from clopidogrel and low doses of aspirin depends on CYP2C19 genotype. Clin. Gastroenterol. Hepatol..

[30] Abdelkader N.A., Fouad Y., Shamkh M.A., Elnabawy O.M., Eid R.A., Attia D., Abdeltawab D., Khalil N.O., Abdallah M., Abdelhalim S.M. (2025). Prevalence of spontaneous fungal peritonitis in Egyptian cirrhotic patients with ascites. Egypt. Liver J..

[31] Ng F., Wong S., Chang C., Chen W., Kng C., Lanas A., Wong B. (2003). High incidence of clopidogrel-associated gastrointestinal bleeding in patients with previous peptic ulcer disease. Aliment. Pharmacol. Ther..

[32] Mihalkanin L., Stancak B. (2020). The impact of novel anticoagulants on the upper gastrointestinal tract mucosa. Medicina.

[33] Dakroub A., Beaini H., Kibbi R., Moumneh M.B., Halablab S.M., Dankar R., Adra N., Rizk C., Barada K., Refaat M. (2024). Comparative Analysis of Anticoagulation Versus Combination Anticoagulation and Antiplatelet Therapy in Atrial Fibrillation Patients Presenting With Gastrointestinal Bleeding. J. Cardiovasc. Pharmacol..

[34] van Diepen S., Fuster V., Verma S., Hamza T.H., Siami F.S., Goodman S.G., Farkouh M.E. (2017). Dual antiplatelet therapy versus aspirin monotherapy in diabetics with multivessel disease undergoing CABG: FREEDOM insights. J. Am. Coll. Cardiol..

[35] Davis A., Robson J. (2016). The dangers of NSAIDs: Look both ways. Br. J. Gen. Pract..

[36] Aoki T., Nagata N., Niikura R., Shimbo T., Tanaka S., Sekine K., Kishida Y., Watanabe K., Sakurai T., Yokoi C. (2015). Recurrence and mortality among patients hospitalized for acute lower gastrointestinal bleeding. Clin. Gastroenterol. Hepatol..

[37] Yen H.-H., Wu P.-Y., Wu T.-L., Huang S.-P., Chen Y.-Y., Chen M.-F., Lin W.-C., Tsai C.-L., Lin K.-P. (2022). Forrest classification for bleeding peptic ulcer: A new look at the old endoscopic classification. Diagnostics.

[38] Burke L.G., Frakt A.B., Khullar D., Orav E.J., Jha A.K. (2017). Association between teaching status and mortality in US hospitals. Jama.

[39] Eid R.A., Abdel Fattah A.M., Haseeb A.F., Hamed A.M., Shaker M.A. (2024). Percutaneous radiofrequency ablation for stage B1 of modified Bolondi’s subclassification for intermediate-stage hepatocellular carcinoma. Egypt. Liver J..

[40] Holster I.L., Kuipers E.J. (2011). Update on the endoscopic management of peptic ulcer bleeding. Curr. Gastroenterol. Rep..

[41] Oakland K., Kahan B.C., Guizzetti L., Martel M., Bryant R.V., Brahmania M., Singh S., Nguyen N.Q., Sey M.S.L., Barkun A. (2019). Development, validation, and comparative assessment of an international scoring system to determine risk of upper gastrointestinal bleeding. Clin. Gastroenterol. Hepatol..

[42] Stanley A.J., Dalton H.R., Blatchford O., Ashley D., Mowat C., Cahill A., Gaya D.R., Thompson E., Warshow U., Hare N. (2011). Multicentre comparison of the Glasgow Blatchford and Rockall scores in the prediction of clinical end-points after upper gastrointestinal haemorrhage. Aliment. Pharmacol. Ther..

[43] Gu L., Xu F., Yuan J. (2018). Comparison of AIMS65, Glasgow–Blatchford and Rockall scoring approaches in predicting the risk of in-hospital death among emergency hospitalized patients with upper gastrointestinal bleeding: A retrospective observational study in Nanjing, China. BMC Gastroenterol..

[44] Liu S., Zhang X., Walline J.H., Yu X., Zhu H. (2021). Comparing the performance of the ABC, AIMS65, GBS, and pRS scores in predicting 90-day mortality or rebleeding among emergency department patients with acute upper gastrointestinal bleeding: A prospective multicenter study. J. Transl. Intern. Med..

[45] Chang A., Ouejiaraphant C., Akarapatima K., Rattanasupa A., Prachayakul V. (2021). Prospective comparison of the AIMS65 score, Glasgow-Blatchford score, and Rockall score for predicting clinical outcomes in patients with variceal and nonvariceal upper gastrointestinal bleeding. Clin. Endosc..

[46] Nakamura S., Matsumoto T., Sugimori H., Esaki M., Kitazono T., Hashizume M. (2014). Emergency endoscopy for acute gastrointestinal bleeding: P rognostic value of endoscopic hemostasis and the AIMS65 score in J apanese patients. Dig. Endosc..

[47] Yang H.M., Jeon S.W., Jung J.T., Lee D.W., Ha C.Y., Park K.S., Lee S.H., Yang C.H., Park J.H., Park Y.S. (2016). Comparison of scoring systems for nonvariceal upper gastrointestinal bleeding: A multicenter prospective cohort study. J. Gastroenterol. Hepatol..

[48] Budimir I., Stojsavljević S., Baršić N., Bišćanin A., Mirošević G., Bohnec S., Kirigin L.S., Pavić T., Ljubičić N. (2017). Scoring systems for peptic ulcer bleeding: Which one to use?. World J. Gastroenterol..

[49] Ramaekers R., Mukarram M., Smith C.A., Thiruganasambandamoorthy V. (2016). The predictive value of preendoscopic risk scores to predict adverse outcomes in emergency department patients with upper gastrointestinal bleeding: A systematic review. Acad. Emerg. Med..

[50] Laursen S.B., Dalton H.R., Murray I.A., Michell N., Johnston M.R., Schultz M., Hansen J.M., de Muckadell O.B.S., Blatchford O., Stanley A.J. (2015). Performance of new thresholds of the Glasgow Blatchford score in managing patients with upper gastrointestinal bleeding. Clin. Gastroenterol. Hepatol..

[51] González González J.A., Vázquez Elizondo G., García Compeán D., Obed Gaytán Torres J., Flores Rendón Á.R., Jáquez Quintana J.O., Garza Galindo A.A., Cárdenas Sandoval M.G., Maldonado Garza H.J. (2011). Predictors of in-hospital mortality in patients with non-variceal upper gastrointestinal bleeding. Rev. Española Enfermedades Dig..

[52] In K.R., Oh Y.E., Moon H.S., Jung S., Kang S.H., Sung J.K., Jeong H.Y. (2024). Comparison and validation of several scoring systems for non-variceal upper gastrointestinal bleeding: A retrospective study. Sci. Rep..

[53] Abd El-Ghani S.E.-S., Hamed R.M.R., Eid R.A., Ibrahim A.Y.M., Abdel-Hamid H.M., Abdelrahman W., Ibrahim R.E., Abdel-Aziz M.M., Mohamed M.S. (2022). Serum interleukin 1β and sP-selectin as biomarkers of inflammation and thrombosis, could they be predictors of disease severity in COVID 19 Egyptian patients? (a cross-sectional study). Thromb. J..

[54] Camellini L., Merighi A., Pagnini C., Azzolini F., Guazzetti S., Scarcelli A., Manenti F., Rigo G. (2004). Comparison of three different risk scoring systems in non-variceal upper gastrointestinal bleeding. Dig. Liver Dis..

[55] Jeon H.J., Moon H.S., Kwon I.S., Kang S.H., Sung J.K., Jeong H.Y. (2021). Which scoring system should be used for non-variceal upper gastrointestinal bleeding? Old or new?. J. Gastroenterol. Hepatol..

[56] Castiello D.S., Oliva A., Andò G., Niccoli G., Pelliccia F., Moscarella E., Montone R.A., Gragnano F., Porto I., Calabrò P. (2025). Antithrombotic therapy in complex percutaneous coronary intervention. EuroInterv. J. Eur. Collab. Work. Group Interv. Cardiol. Eur. Soc. Cardiol..

